# Characteristics of Different Types of Basic Oxygen Furnace Slag Filler and its Influence on Properties of Asphalt Mastic

**DOI:** 10.3390/ma12244034

**Published:** 2019-12-04

**Authors:** Dezhi Kong, Shaopeng Wu, Meizhu Chen, Meiling Zhao, Benan Shu

**Affiliations:** 1 State Key Laboratory of Silicate Materials for Architectures, Wuhan University of Technology, Wuhan 430070, China; kongdz@whut.edu.cn (D.K.); wusp@whut.edu.cn (S.W.); shuba@whut.edu.cn (B.S.); 2 Research Institute of Highway of Ministry of Transport, Beijing 100088, China; zhaoml@whut.edu.cn

**Keywords:** BOF slag filler, asphalt mastic, morphological characteristics, rheological properties

## Abstract

The fillers of ordinary and pyrolytic basic oxygen furnace (BOF) slag were selected to investigate the properties of their asphalt mastic. XRF (X-Ray Fluorescence) was used to analyze chemical composition of fillers. Meanwhile, SEM (Scanning Electron Microscope) and AIMS (Aggregate Image Measurement System) were utilized to explore meso-morphology, angularity and sphericity. Penetration, softening point and viscosity of asphalt mastic were discussed, while the rheological properties of asphalt mastic were studied by means of DSR (dynamic shear rheometer) and BBR (bending beam rheometer) tests. The experimental results show that chemical composition of different types of BOF slag is similar. The grinding energy consumption of pyrolytic BOF slag is higher than that of limestone and ordinary BOF slag. It is not recommended that pyrolytic BOF slag filler is produced by grinding process. The micro-texture structure of ordinary BOF slag filler is more abundant and their angularity index is about 15% higher than that of limestone filler. The stiffness modulus and rutting factor of asphalt mastic with ordinary BOF slag filler is higher than that of limestone filler. Meanwhile the incorporation of BOF slag filler will further reduce the low-temperature flow performance of asphalt mastic. The effect of pyrolytic BOF slag filler on the performance of asphalt mastic is less than that of ordinary BOF slag. Ordinary BOF slag filler can effectively improve high temperature anti-rutting stability of asphalt mixture. Ordinary BOF slag has a useful application prospect as filler in asphalt mixture.

## 1. Introduction

Steel slag is a common by-product of the steelmaking industry, and its output is about 10% to 15% of steel production in the world [1,2,3]. As a major type of steel slag, basic oxygen furnace (BOF) slag has strong alkalinity, rich angularity, tough surface characteristics and relatively good mechanical properties [4]. BOF slag is widely used as aggregate in asphalt mixtures in related research [5,6,7]. Pasetto et al. and Wu et al. [8,9,10] demonstrated that BOF slag aggregates improve performance of asphalt mixtures, such as moisture stability, high temperature deformation resistance, abrasion and skid resistance. BOF slag is an ideal substitute for natural aggregates in asphalt mixture. 

Asphalt mixtures consist of asphalt binder, aggregate, and mineral filler. Asphalt mastic, which refers to mixture of asphalt binder and mineral filler, determines the final mechanical properties of asphalt mixture. Previous studies [11,12] have demonstrated that the properties of asphalt mastic are the factors influencing the rutting resistance and low-temperature crack resistance of asphalt mixtures, while the properties of filler are closely associated with the asphalt mastic. Many researchers [13,14,15,16,17] have proved that performances of asphalt mastic are affected by the volume content of fillers and performances of fillers like surface characteristics, alkalinity and size, as well as physical-chemical interaction between asphalt and fillers. 

Xiao et al [18,19] discussed the feasibility of BOF slag as mineral filler in asphalt mixtures and the results show that the asphalt mastic with BOF slag filler has better high-temperature rheological properties than that of limestone filler. Qiu et al [20] examined the low-temperature fracture properties of asphalt mastic using steel slag powder. The results showed that the steel slag powder–asphalt system had higher fracture resistance than conventional systems and steel slag powder can diminish the severity of low-temperature reversible aging of modified asphalt. Song et al [21] demonstrated that the steel-making dust would be an alternative to the ordinary mineral filler to improve the performance of asphalt mortars and reduce the harm of the dust to the environment at the same time.

With the rapid development of highway construction projects and the consequential deterioration in high-quality mineral filler, there is an urgent need to broaden the source of the fillers that can be used in asphalt mixtures. Meanwhile, lots of numerous micro fillers are produced in the crushing process and magnetic separation of BOF slag [22]. The utilization of BOF slag as filler in asphalt mixture has attracted more concern. Furthermore, different types of BOF slag have different properties. Meanwhile, the difference in physicochemical properties of fillers and their influence on asphalt mastic is still unknown.

**Figure 1:**
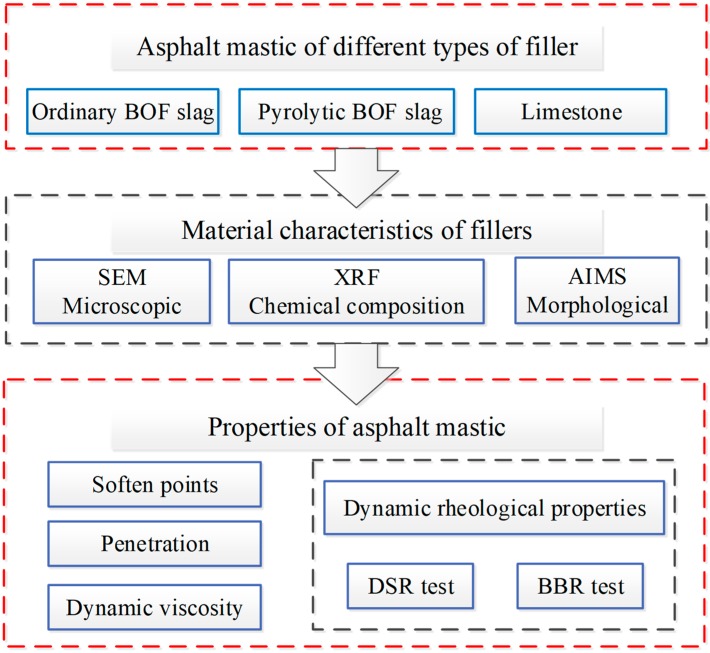
Finalized research program.

This study attempted to evaluate the feasibility of different types of BOF slag used as mineral filler to replace limestone filler (LF) in asphalt mixtures. All types of fillers were obtained by grinding 3–5 mm particle size range aggregates with the same processing conditions. It was found that 90% of all types of fillers could pass through 0.075 mm sieve. Figure 1 illustrates the research program on the characteristics of different types of BOF slag filler and their influence on properties of asphalt mastic. Firstly, chemical composition of different types of BOF slag filler was evaluated by XRF. Secondly, the geometrical properties of BOF slag filler were examined, such as meso-morphology and angularity and sphericity. Thirdly, the basic physical properties of asphalt mastic were discussed including penetration, softening point and viscosity. Finally, the rheological properties of asphalt mastic were studied at both low and high temperatures.

## 2. Materials and Methods

### 2.1. Raw Materials

Pen 60/80 bitumen binder, provided by Panjin Co., Ltd., Liaoning, Panjin, China, was used in this research. The basic properties of bitumen binder are shown in Table 1. They are all within the requirement specifications of China.

Three types of BOF slag fillers and limestone filler were employed in this research. The limestone (L) was provided by Hubei province in China. Type A BOF slag (BS-A) was obtained from ironworks in Hubei province in China, while Type B BOF slag (BS-B) and pyrolytic BOF slag (PBS) were obtained from ironworks of Baotou in Inner Mongolia, China. BS-A and BS-B were basic oxygen furnace slag, and PBS was pyrolytic BOF slag which was treated with a hot stuffing process during the BOF slag cooling step. 

All types of fillers in this research were obtained by grinding 3–5 mm particle size range aggregates with the same processing conditions. A ball mill was used and the time of the grinding was 60 min, 5,000 g for each sample and the rotating speed was 120 r/min. After grinding, more than 90% of all types of fillers could pass through the 0.075 mm sieve. The labels are as follows: limestone filler (LF), type A BOF slag filler (BSF-A), type B BOF slag filler (BSF-B), pyrolytic BOF slag filler (PBSF).

The basic properties of the four types of fillers are shown in Table 2. It can be deduced that the density of BOF slag is higher than that of LF. The density of BSF-A and BSF-B is almost equal, while they are about 18% higher than that of LF. Among them, the density of PBSF is the highest, which is attributed to the higher content of Fe_2_O_3_ and the hot stuffing process during BOF slag cooling step. The difference in hydrophilic coefficient of the four fillers is virtually negligible. However, the water absorption of BOF slag fillers is higher than that of LF, which is due to the particular pore structure of the surface of BOF slag.

### 2.2. Experimental Methods 

#### 2.2.1. Properties of Fillers

The chemical compositions of the four types of filler were evaluated by X-Ray Fluorescence (PANalytical. B.V., Zetium, Almelo, Netherlands). The surface characteristics of four types of fillers were evaluated using scanning electron microscope (JSM-IT300, SEM-JEOL, Tokyo, Japan). The aggregate image measurement system (AIMS) was used to analyze the morphological features of each type of filler. Meanwhile, the laser particle size analyzer (Mastersizer-2000, Malvern, England) was utilized to analyze the difference of particle size of four types of fillers.

#### 2.2.2. Preparation of Asphalt Mastic 

Specific amounts of each type of filler were incorporated into pen 60/80 asphalt binder to prepare the asphalt mastic. Firstly, asphalt binder was preheated to 150 °C for 30 min. Then, filler was gradually incorporated with a high shear instrument of the shear speed of 4000 rpm for 30 min. Homogeneous dispersion of the filler in the asphalt binder was required for further research. the filler–asphalt volume ratio of asphalt mastic used in this research was 0.4. The mass–volume conversions of four fillers were calculated by the density values in Table 2. After calculation, the filler-asphalt weight ratios of LF was 1.113, BSF-A was 1.314, BSF-B was 1.32, and PBSF was 1.42.

#### 2.2.3. Properties of Asphalt Mastic

The penetration and softening point test were used to assess the basic properties of the asphalt mastic. In the preparation process of asphalt mixture, the workability is closely linked to the viscosity of asphalt mastic. A Brickfield viscometer was used to analyze the difference of four types of asphalt mastic. Test methods of penetration, softening point and dynamic viscosity refer to Chinese official standard JTG E20-2011 [23]. 

A dynamic shear rheometer (MCR101, DSR, Anton Paar, Graz, Austria) was utilized to evaluate the rheological properties of asphalt mastics. The DSR test was performed at a fixed frequency of 10 rad/s under variation of temperature from −10 °C to 80 °C with increments of 2 °C/min. In −10 °C to 3 °C, specimens were placed on a parallel plate geometry whose diameter was 8 mm, the thickness was 2 mm and strain level was 0.2%. In 30 °C to 80 °C, specimens were placed on a parallel plate geometry whose diameter was 25 mm, the thickness was 1 mm and strain level was 2.0%. The BBR (TE-BBR, Cannon, New York, NY, USA) was used to examine the rheological properties at a low temperature, which relates to the low-temperature cracking resistance. Preheated asphalt mastic was filled into an aluminum mold to prepare a mastic beam 102.0 ± 0.5 mm in length, 12.7 ± 0.5 mm in width, and 6.25 ± 0.5 mm in thickness. Drawing on the research of Xiao et al [18,19], tests were performed at a definite temperature (15 °C). Specimens were tested under a constant stress of 0.985 N for 250 s. Each test for different mastic included five duplicate specimens and the average value was adopted.

## 3. Results and Discussion

### 3.1. Material Characteristics of Fillers

#### 3.1.1. Chemical Compositions of Fillers 

The chemical compositions of the four types of filler from X-ray fluorescence analysis are shown in Table 3. Limestone is an alkali aggregate because the chemical composition of CaO was higher than fifty percent. The chemical composition of Fe_2_O_3_ in three types of BOF slags is more than 20%. Three types of BOF slag contain less SiO_2_ and more than 30% CaO making them alkali aggregates.

#### 3.1.2. Microscopic Characteristics of Fillers 

**Figure 2:**
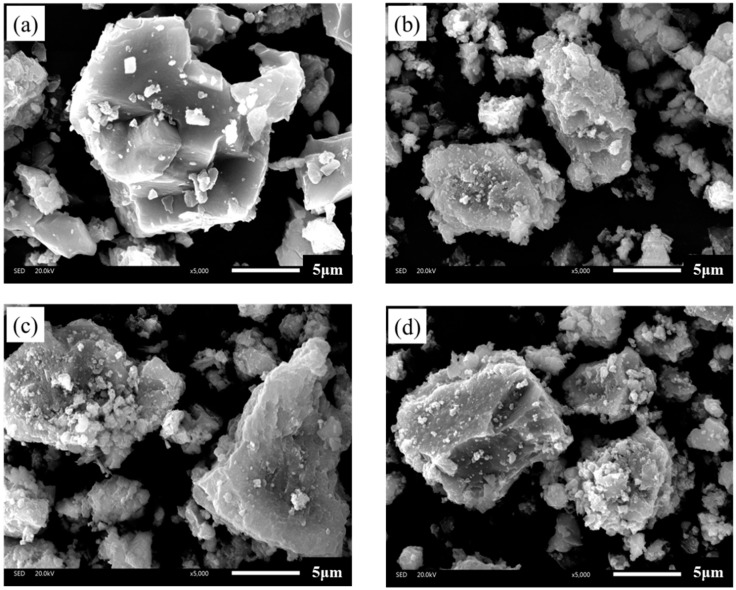
SEM images of investigated fillers: (**a**) limestone filler; (**b**) type A basic oxygen furnace (BOF) slag filler; (**c**) type B BOF slag filler; and (**d**) pyrolytic BOF slag filler.

Figure 2 shows the diversity between limestone filler and three types of BOF slag fillers in SEM images. Compared with the micrographs of LF, BOF slag has different surface texture particularly due to the size and number of numerous tiny particles which are adhered to its surface s and its rough surface. The surface of LF is relatively smoother than that of BOF slag filler, where the latter is irregular shaped, rough, and bumpy, which might result in effective cohesion with asphalt binder and consequently lead to improved strength and water resistance. From practical viewpoint, the rough surface of BOF slag filler will cause an increase in the amount of asphalt required when BOF slag filler is used as filler in asphalt mixtures. 

#### 3.1.3. Morphological Characteristics of Fillers 

Morphological characteristics of fillers in this research were analyzed by AIMS, which is an integrated system comprising image acquisition hardware and a computer. Analysis of filler includes gradient angularity (the AIMS Angularity Index ranges from 1 to 10,000) and Form2D (AIMS Form2D Index ranges from 0 to 20).

Angularity is a description of edge sharpness of the boundary particles of aggregate. The angularity changes with filler granule boundary shape changes. The value of angularity is calculated based on the gradient on the particle boundary. Angularity is calculated with Equation (1) [24]. Its range is 0 to 10,000. The larger the value of angularity is, the boundary shape of filler is sharper.

(1) $$Gradient\;Angularity=\frac{1}{\frac{n}{3}-1}\overset{n-3}{\underset{i=1}{\sum }}\left|\theta_{i}-\theta_{i+3}\right|$$

where *θ* is angle of orientation of the edge points, *n* is the total number of points, *i* is the ith point on the edge of the particle.

**Figure 3:**
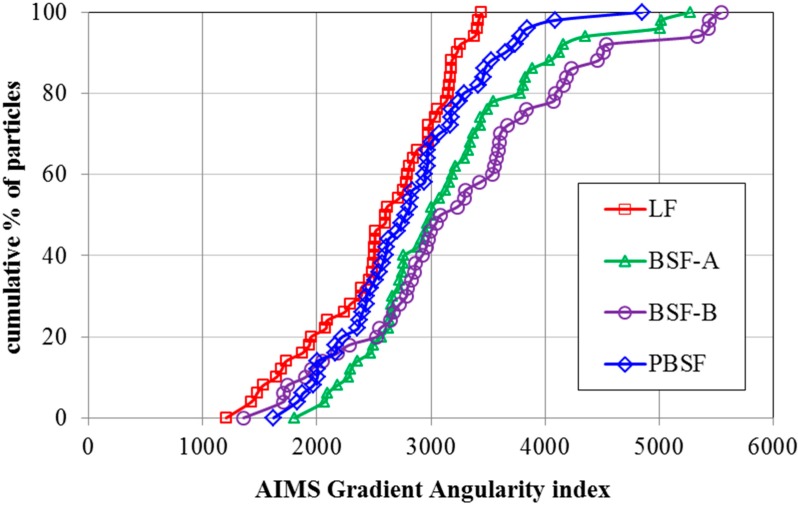
Comparison of distributions of angularity indexes of four types of fillers.

Figure 3 shows the comparison of distributions of gradient angularity indexes of four types of fillers. It can be clearly seen that LF has the lowest gradient angularity indexes while BSF-B has the highest gradient angularity indexes. The distribution range of the angularity index of LF is narrower than that of BSF-A, BSF-B and PBSF. Such differences illustrate that the distribution of gradient angularity index of LF is more uniform. By comparing three types of BOF slag filler, it can be found that the gradient angularity index of PBSF is smaller than that of ordinary BOF slag. The gradient angularity index of ordinary BOF slag is about 15% higher than that of LF. In summary, the particle shape of the BOF slag filler has more angular structure than that of limestone filler under the same grinding process conditions.

AIMS Form2D is applicable only to fine-sized aggregate and it quantifies the relative form from 2D images of aggregate particles. Form2D is calculated with Equation (2) [25] and its range is 0 to 20. A perfect circle has a Form2D value of 0. According to the definition of Form2D, a higher Form2D value would imply a relatively rougher surface, which would consequently indicate a positive contribution towards adhesive mechanical bonding of asphalt binder to filler.

(2) $$Form\;2D=\overset{\theta =360-\Delta \theta }{\underset{\theta =0}{\sum }}\left[\frac{R_{\theta +\Delta \theta }-R_{\theta }}{R_{\theta }}\right]$$

where *R_θ_* is the radius of the particle at an angle of *θ*, Δ*θ* is the incremental difference in the angle.

**Figure 4:**
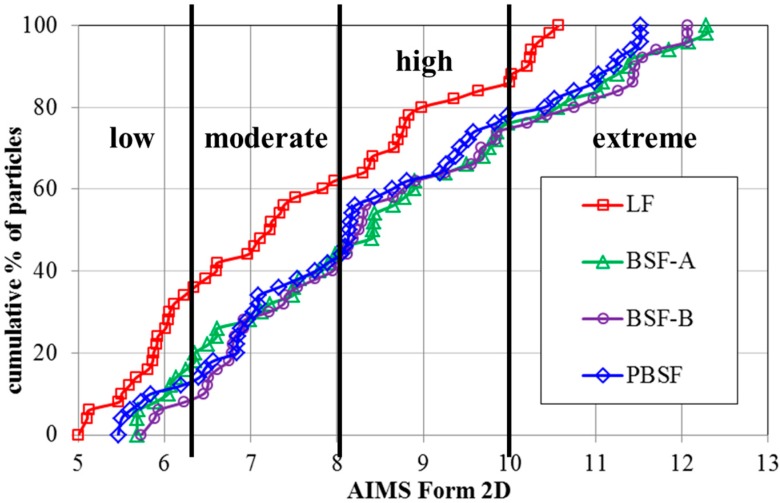
Distributions of Form2D values of four types of fillers.

As shown in Figure 4, the Form2D values of four types of fillers have a wide distribution range of 5–12. For each type of BSF, nearly 80% of the particles have moderate and higher Form 2D values while about 40% of LF has lower Form 2D values. The Form2D values of LF is the lowest and the Form 2D of BOF slag is about 10% higher than that of LF. In summary, the surface of BOF slag filler was rougher and sharper than that of limestone filler. These results agree with the SEM results.

#### 3.1.4. Particle Size Analysis of Fillers 

**Figure 5:**
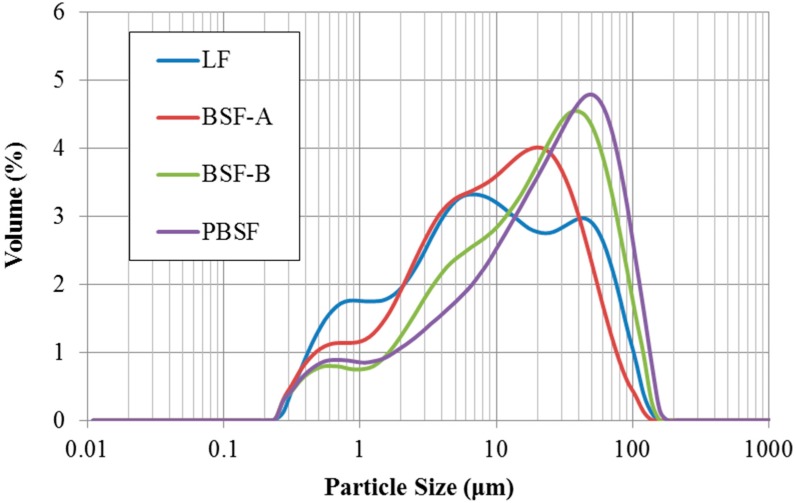
The particle size distribution of four types of filler.

Figure 5 shows the particle size distribution of four types of filler. It reveals that the particle size of the four types of fillers is different under the same grinding process conditions. The particle size of LF is minimal, while the particle size of PBSF is maximal. Slight differences exist between LF and BSF-A in particle size. The order of particle size from small to large is LF, BSF-A, BSF-B and PBSF. The grinding efficiency of BOF filler is relatively lower than that of LF. The grinding efficiency of different kinds of BOF filler is also different. Pyrolytic BOF slag has the maximum particle size among the four types of fillers, indicating that pyrolytic BOF slag is more difficult to grind than ordinary BOF slag. In order to achieve the same particle size, the grinding process of pyrolytic BOF slag needs to consume more grinding time and more energy consumption.

### 3.2. Properties of Asphalt Mastic

Four types of asphalt mastics and basic bitumen binder were investigated in this research. Both basic properties and dynamic rheological properties were analyzed.

#### 3.2.1. Basic Properties of Asphalt Mastic

The basic properties of asphalt mastic investigated in this research include soften points, penetration and dynamic viscosity. 

The bitumen binder has a typical viscosity property. The smaller the penetration value is, the greater the viscosity of the asphalt material under low temperature conditions is, and the corresponding elastic deformation performance and recovery performance are also better. The softening point of the asphalt mastic is larger, indicating that the high temperature performance is better.

In Table 4 the values of soften points and penetration of asphalt mastic are presented. Compared with LF asphalt mastic, the softening point of asphalt mastic with BOF slag filler is lower, while its penetration is higher. BOF slag filler has a better viscosity-increasing effect on asphalt mastic than LF. The softening point and penetration of asphalt mastic with three types of BOF slag filler are similar. Although the PBSF has the largest particle size, its effect on softening point increase and penetration reduction is still effective. The particle size factor and morphological characteristics of BOF slag filler has little effect on the soften points and penetration of asphalt mastic.

The dynamic viscosity of asphalt is very important for asphalt mixture. The reasonable construction temperature is determined by the dynamic viscosity temperature range of 0.17 Pa s ± 0.02 Pa s, and construction rolling temperature is determined by the dynamic viscosity temperature range of 0.28 Pa s ± 0.03 Pa s [26]. The consistency of asphalt mastic in asphalt mixture construction is deeply influenced by the dynamic viscosity [27].

Viscosity–temperature value for bitumen binder and four types of asphalt mastic are shown in Table 5. In the case of limestone filler, the viscosity of asphalt mastic at different temperatures is approximately five times higher than that of bitumen binder. The addition of filler can effectively improve the viscosity of asphalt mastic. Compared with LF asphalt mastic, the viscosity of BSF asphalt mastic is higher, mainly because BOF slag filler has higher alkalinity and rougher micro-texture. The dynamic viscosity of the three types of asphalt mastic from large to small is BSF-A, BSF-B and PBSF. It is believed that the bigger particle size, more regular shape and smoother microscopic surface of PBSF result in a relatively lower dynamic viscosity of asphalt mastic.

#### 3.2.2. Dynamic Rheological Properties of Asphalt Mastic

As a typical viscoelastic material, the dynamic rheological properties of asphalt are closely related to its load and temperature conditions [28]. The viscoelastic characteristics of different types of asphalt and asphalt mastic are complex in different temperature conditions. Dynamic shear rheometer (DSR) tests were used to study dynamic rheological properties of asphalt mastic in this research. Complex shear modulus (G*) and phase angle (δ) are recorded and calculated by performing the DSR test at varying temperatures. G* can be decomposed into storage shear modulus (G’ = G*cosδ) and loss shear modulus (G′′ = G*sinδ), which is used to characterize the ability of asphalt mastic to resist deformation under repeated shear loads. The larger of the G* is, the higher of the resistance of asphalt mastic is in deformation. δ is the time lag of the applied stress and the resulting strain. The tangent value of δ is the ratio of the loss modulus to the storage modulus. A smaller δ indicates that there are more elastic components in the asphalt G*, and a larger δ indicates that there are more viscous components in the asphalt G*. For a fully elastic material, the phase angle δ is zero, at which point all deformations are recoverable. However, for viscous materials, the phase angle is close to 90°, at which point all deformation is permanent. G*/sinδ (the ratio of the complex modulus to phase angle sine) characterizes the ability of asphalt to resist high temperature rutting. G*/sinδ is called the rutting factor. Under the same temperature conditions, asphalt mastic with larger G*/sinδ has better rutting resistance [29].

**Figure 6:**
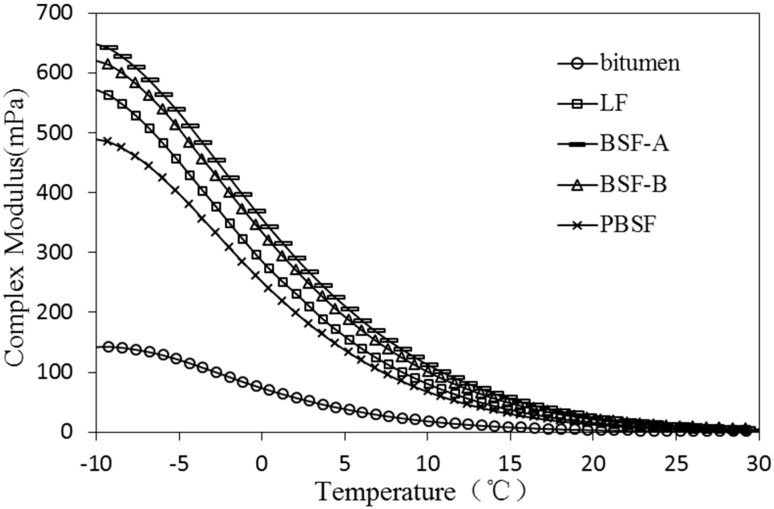
Effect of different fillers on G* of asphalt mastic (−10~30 °C).

**Figure 7:**
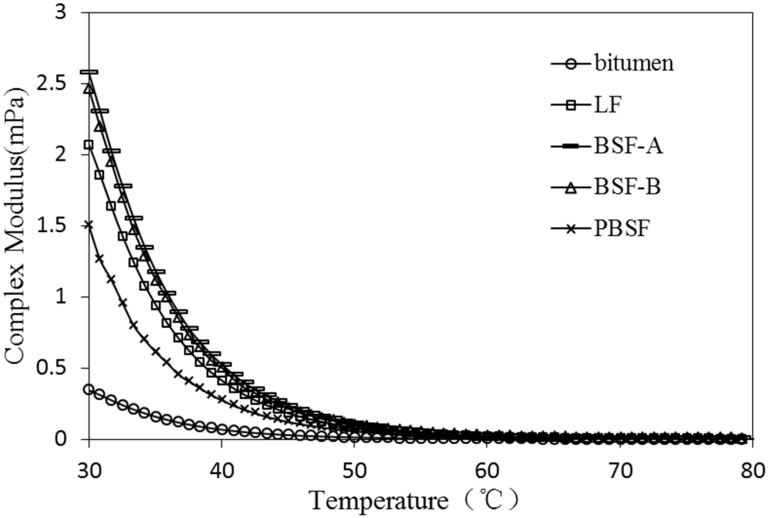
Effect of different fillers on G* of asphalt mastic (30~80 °C).

Effect of different fillers on G* of asphalt mastic are shown in Figure 6 and Figure 7. The G* of the asphalt mastic and asphalt decreases with the increase of temperature, which indicates that the rheological properties of the asphalt are greatly affected by the temperature. When the temperature rises, the volume of free asphalt increases, and the pitch changes from a high elastic state at a low temperature to a viscous state at a high temperature. As a result, the maximum shear stress and the maximum shear strain of the asphalt are increased when the shear force is applied, and therefore the G* is lowered.

In the temperature range of −10~30 °C, the higher the G* of the asphalt mastic is, the better the crack resistance of the asphalt under low temperature conditions. As shown in Figure 6, the values of complex modulus of BSFA are highest and PBSF has the worst effect on the increase of complex modulus. The values of PBSF are lower than that of LF, mainly due to a small alkalinity, a large particle size, a large specific surface area, less adsorbed asphalt, and relatively more free asphalt. Ordinary BOF slag filler has a good effect, improving the low temperature crack resistance of asphalt mastic.

In Figure 7, the effect of different fillers on G* of asphalt mastic (30~80 °C) is presented. After adding BSF-A and BSF-B, the G* of asphalt mastic is higher than that of LF, which indicates that the high temperature stability of ordinary BOF slag filler asphalt mastic is better than that of LF.

**Figure 8:**
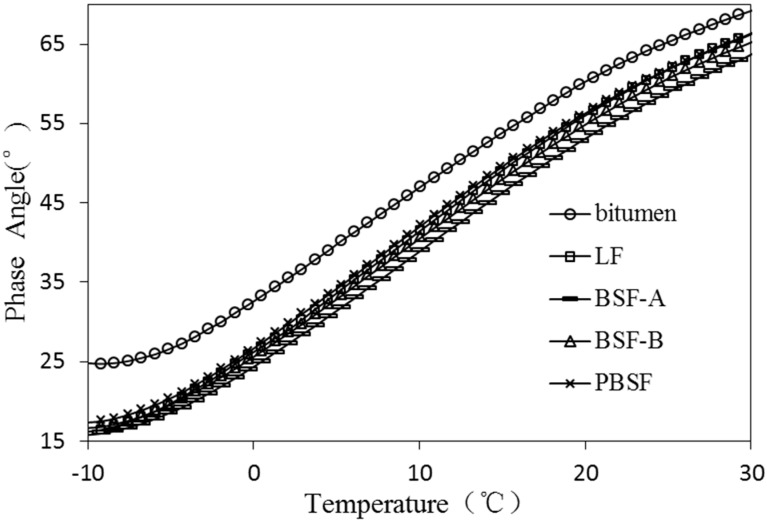
Effect of different fillers on δ of asphalt mastic (−10~30 °C).

**Figure 9:**
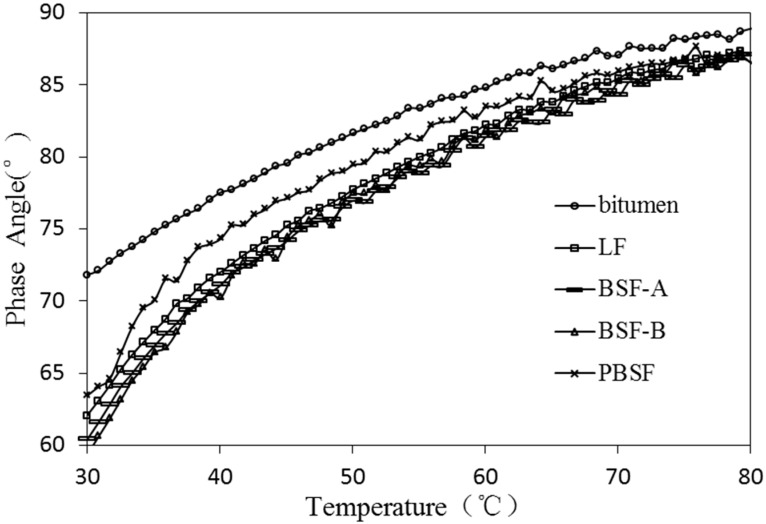
Effect of different fillers on δ of asphalt mastic (30~80 °C).

In Figure 8 and Figure 9 the values of different fillers on δ of asphalt mastic (−10~80 °C) are presented. The δ of asphalt mastic with different types of fillers increases with the increase of temperature, and the difference of δ values of asphalt mastic decreases with the increase of temperature. The sensitivity of the δ of the filler is continuously reduced with the temperature increasing. The phase angles of asphalt mastics with different types of fillers are arranged from large to small in order, pure asphalt, PBSF, LF, BSF-B and BSF-A. It is shown that ordinary BOF slag can effectively improve the elastic properties of asphalt mastic, and is beneficial, improving the anti-permanent deformation performance of asphalt mastic.

**Figure 10:**
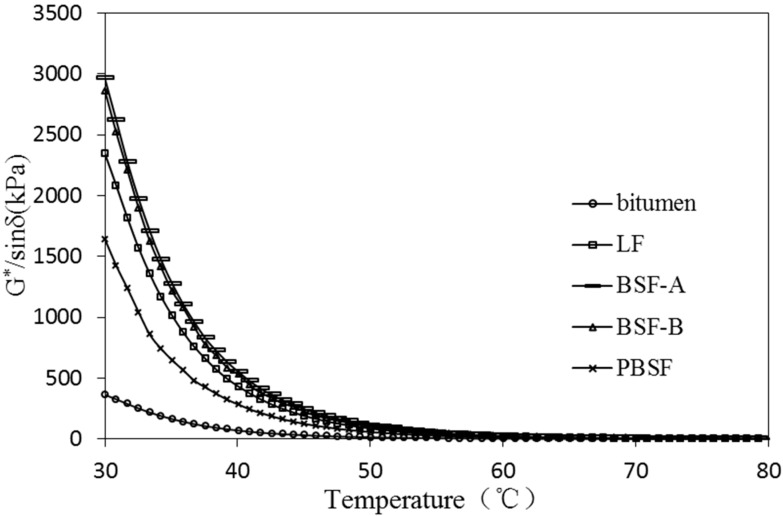
Effect of different fillers on G*/sinδ of asphalt mastic (30~80 °C).

As shown in Figure 10, the rutting factor of the asphalt mastics with BSF-A and BSF-B are similar and larger than that of corresponding PBSF asphalt mastic. It demonstrates that all asphalt mastics containing ordinary BOF slag fillers have better high-temperature deformation resistance than ones with LF. BSF mastic presents the best deformation resistance. This was due to the chemical effect between alkaline components in ordinary BOF slag fillers and asphaltic acid in bitumen. The stiffness of BOF slag makes mastic structure more stable to resist permanent deformation.

#### 3.2.3. Anti-Cracking Properties of Asphalt Mastic at Low Temperature

Bending beam rheometer (BBR) testing was used to investigate the low-temperature rheological properties of asphalt mastic in this research. Tests were performed at a fixed temperature (−15 °C) to discuss the different of low temperature performance of asphalt mastic with different filler, meanwhile the m-value and creep stiffness (S(t), MPa) were evaluated. The S(t) was calculated on the basis of Equation (3). The m-value signifies the rate that S(t) changes during loading time. Creep stiffness indicates the thermal stress of asphalt mastic under low temperature. Lower creep stiffness implies that the specimen has better rheological properties at lower temperatures. Scientifically, lower creep stiffness is positive because it corresponds to lower deformation stress. M-value reflects the stress relaxation property of asphalt mastic at low temperatures. A higher m-value is required since asphalt with a higher m-value has better ability to disperse deformation stress [30].

(3) $$S\left(t\right)=\frac{PL^{3}}{4bh^{3}\Delta (t)}$$

where *b*, *h* and *L* are the width (mm), height (mm), length (mm) of specimen. *P* is the constant applied load. Δ(*t*) is the deflection of beam (mm) at different times (*t*).

**Figure 11:**
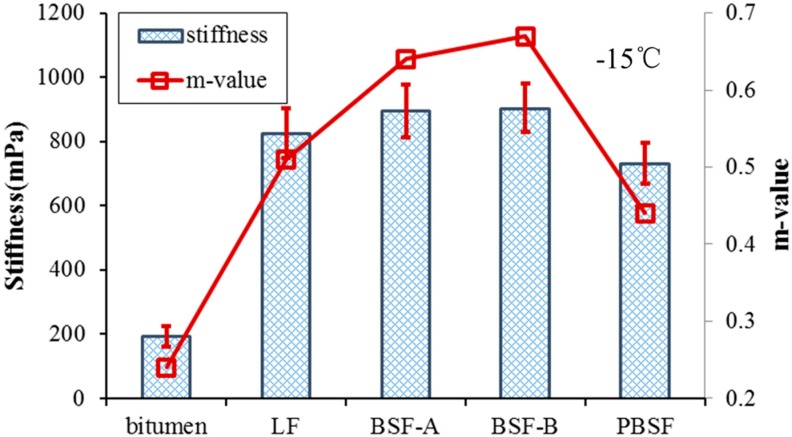
Bending beam rheometer (BBR) test results of four asphalt mastic and pure bitumen.

The creep stiffness and m-value of asphalt mastic with different types of filler are shown in Figure 11. The change of m-value is similar to the stiffness. It can be clearly seen that the introduction of fillers increases the *S*(*t*) and m-value. The *S*(*t*) values of LF are about four times that of pure asphalt, so asphalt will become stiffer at a low temperature after mixing with fillers. Considering the value of stiffness, the stiffness of PBSF is smaller than that of LF, which indicates that pyrolytic BOF slag filler has a positive effect on the low-temperature crack resistance performance of asphalt mastic. Asphalt mastics with BSF-A and BSF-B are higher than ones with LF. It indicates that ordinary BOF slag filler has a certain increase in the stiffness of asphalt mastic, but has a negative influence on its low-temperature rheological properties. The incorporation of ordinary BOF slag filler reduces the low-temperature crack resistance performance of asphalt mastic compared with LF. 

## 4. Conclusions

The microscopic characteristics, morphological characteristics (angularity and Form2D) and chemical properties of three types of BOF slag filler were investigated in the first part of this research. Then the basic physical properties and rheological properties of their asphalt mastic were studied. The overall conclusions are elaborated upon hereunder:

(1) The chemical composition of BOF slag is more complicated than that of limestone, which includes SiO_2_, CaO, MgO, Al_2_O_3_, Fe_2_O_3_ and other components. The chemical composition of different types of BOF slag is similar. The micro-texture structure of BOF slag filler is more complex than that of LF. The angularity index of ordinary BOF slag is about 15% higher than that of LF. The angularity index, Form2D and micro texture of different types of BOF slag filler are also different. 

(2) The asphalt mastic with BOF slag has higher soften points, lower penetration and higher dynamic viscosity than one with LF. The incorporation of BOF slag filler can significantly improve the high-temperature stability of asphalt mastic. 

(3) Compared with rheological properties, asphalt mastic with BOF slag filler has higher stiffness modulus and rutting factor than that of LF asphalt mastic. The effect of pyrolytic BOF slag filler on the performance of asphalt mastic is less than that of ordinary BOF slag because of the bigger particle size, more regular shape and relatively clean surface. The incorporation of BOF slag filler will reduce the low temperature flow performance of asphalt. 

(4) The grinding energy consumption of pyrolytic BOF slag is higher than that of limestone and ordinary BOF slag, meanwhile its chemical performance is relatively inactive. It is not recommended that pyrolytic BOF slag filler is produced by grinding technology. Ordinary BOF slag filler can effectively improve high temperature anti-rutting stability when used as filler in asphalt mixture. BOF slag filler has a good prospects for application as part of asphalt mixtures.

## Figures and Tables

**Table 1:** Basic properties of the bitumen binder in this research.

Properties	Values	Specifications
Penetration [0.1 mm]	64	60–80
Penetration index	−0.6	−1.5–1.0
Softening point [°C]	46.9	≥46
Ductility, 5 cm/min, 15 °C [cm]	167	≥100
Dynamic viscosity (60 °C) [Pa·s]	172	≥160
Density [g/cm^3^]	1.021	

**Table 2:** Basic properties of four types of fillers.

Property	Density (kg/m^3^)	Hydrophilic Coefficient	Water Absorption (%)
LF	2725	0.64	0.53
BSF-A	3217	0.69	0.67
BSF-B	3244	0.73	0.63
PBSF	3478	0.65	0.64

**Table 3:** Chemical composition of the fillers in this research.

Composition [%]	SiO_2_	CaO	MgO	Al_2_O_3_	Fe_2_O_3_	MnO	P_2_O_5_	Other	LOI
LF	0.86	51.2	2.36	0.85	0.12	0.7	1.02	0.19	42.7
BSF-A	19.2	42.7	5.19	3.25	23.9	1.77	1.41	0.22	2.36
BSF-B	17.7	39.7	5.56	2.91	24.4	4.55	1.68	0.09	3.41
PBSF	15.4	34.4	6.22	1.95	30.8	4.46	2.15	0.16	4.46

**Table 4:** The soften points and penetration of asphalt mastic.

Property	Soften Points (°C)	15 °C Penetration (0.1 mm)	25 °C Penetration (0.1 mm)
LF	55.3	13.5	31.5
BSF-A	58.3	12.1	28.4
BSF-B	57.1	11.9	29.3
PBSF	58.5	12.5	29.8

**Table 5:** The dynamic viscosity test results of asphalt mastic.

Property	Dynamic Viscosity (Pa s)				
	90 °C	105 °C	120 °C	135 °C	150 °C
Bitumen binder	11.80	3.40	1.26	0.51	0.23
LF	52.00	14.25	4.95	1.97	0.94
BSF-A	78.00	22.25	7.25	3.00	1.40
BSF-B	77.00	21.05	7.15	2.80	1.38
PBSF	71.00	19.30	6.82	2.63	1.25
